# New Understandings from the Biophysical Study of the Structure, Dynamics, and Function of Nucleic Acids 2.0

**DOI:** 10.3390/ijms232415822

**Published:** 2022-12-13

**Authors:** Joon-Hwa Lee

**Affiliations:** Department of Chemistry and RINS, Gyeongsang National University, Jinju 52828, Gyeongnam, Republic of Korea; joonhwa@gnu.ac.kr

Nucleic acids play an essential role in all biological processes related to genetic information, such as replication, transcription, translation, repair, and recombination. Biophysical studies of the structures and dynamics of nucleic acids and DNA/RNA-binding proteins are very important for understanding their biological functions. Previously, a Special Issue was published in the *International Journal of Molecular Sciences*, entitled “Biophysical Study of the Structure, Dynamics, and Function of Nucleic Acids”, which contained an editorial and 14 research articles.

This second version of the Special Issue, entitled “Biophysical Study of the Structure, Dynamics, and Function of Nucleic Acids 2.0”, contains six invited research articles. Two articles reported on the application of fluorescence to unique RNA structures [1,2]. Pathogenic CUG and CCUG RNA repeats are associated with myotonic dystrophy type 1 and 2 (DM1 and DM2), respectively [3]. Hassan, Sheng, and coworkers investigated the intermolecular interaction between the CUG repeat RNA and its possible ligand, sisomicin, using the fluorescence technique [1]. This study showed that sisomicin is able to bind and stabilize the folded RNA structure and demonstrated that this fluorescence-based binding assay can be used for high-throughput screening of RNA–ligand interactions [1]. Xue and Liu incorporated the fluorophore and its quencher into full-length adenine riboswitch RNAs [2]. They investigated the dynamics of the riboswitch RNAs in vitro and in cell and used them to measure cellular Mg^2+^ concentrations [2]. They suggested that this technique can be used to probe the dynamic features of the biologically active RNAs in cells and may promote the development of RNA sensors and drugs [2].

This Special Issue also contains two research articles that conducted structural and biophysical studies of DNA-binding proteins and their interaction with target DNA [4,5]. Single-stranded DNA (ssDNA)-binding proteins (SSBs) from hyperthermophilic bacteria, *S. solfataricus*, protect ssDNA from extremely hot environments to avoid damage in their genes [6]. Park’s group determined the solution structure of *S. solfataricus* SSBs (ssoSSBs) using NMR data at a high temperature (50 °C) [4] and compared it with X-ray or NMR structures of various SSBs [7,8,9,10,11,12,13]. This study found that ssoSSBs maintain their ssDNA-binding ability at high temperatures, but exhibit different interactions with ssDNA [4]. In addition, NMR backbone dynamics at high temperatures showed the rigidity of the well-structured region [4]. These structural and dynamic properties of ssoSSBs at high temperatures can provide fundamental insight into new application techniques of thermophile proteins [4]. Distal-less 3 (Dlx3) is a homeobox transcription factor (TF) and plays a crucial role in the development and differentiation process [14,15,16]. Lee and coworkers investigated the salt dependence of the homeobox domain of human Dlx3 (Dlx3-HD) with regard to its DNA-binding activity by using NMR spectroscopy [5]. They demonstrated that Dlx3-HD selectively recognizes its consensus DNA sequences through the α3 helix and L1 loop regions with a unique dynamic feature [5]. This study suggests that the dynamic properties of Dlx3-HD, which can be modulated by varying the salt concentrations, might play an important role in target DNA recognition and be associated with tricho-dento-osseous syndrome [5].

In addition, Anitas reported on the fractal characteristics of DNA sequences studied using the frequency chaos game representation and small-angle scattering technique [17]. This approach provided the structural parameters that can be used to distinguish between the scaling properties of complex hierarchical DNA sequences from *Escherichia coli*, mouse, and human mitochondrion [17]. Dzhimak and coworkers reported on the torque effect with regard to the dynamics of open states in a DNA duplex [18]. The authors demonstrated that the values of the torsion moment required for the appearance of open-state zones are close to the experimentally measured data [18].

Overall, the six contributions that make up this Special Issue provide scientific, conceptual, well-performed experimental approaches and excellent discussions about the structure, dynamics, and function of nucleic acids and nucleic acid-binding proteins.
